# Clinical Utility and Cost Effectiveness of Long-Acting Lipoglycopeptides Used in Deep-Seated Infections among Patients with Social and Economic Barriers to Care

**DOI:** 10.3390/pharmacy10010001

**Published:** 2021-12-23

**Authors:** Kayla Antosz, Majdi N. Al-Hasan, Z. Kevin Lu, Benjamin Tabor, Julie Ann Justo, Alexander Milgrom, Joseph Kohn, P. Brandon Bookstaver

**Affiliations:** 1 Department of Clinical Pharmacy and Outcomes Sciences, University of South Carolina College of Pharmacy, Columbia, SC 29208, USA; lu32@email.sc.edu (Z.K.L.); justoj@cop.sc.edu (J.A.J.); bookstaver@cop.sc.edu (P.B.B.); 2 Prisma Health Midlands, Columbia, SC 29203, USA; majdi.alhasan@uscmed.sc.edu (M.N.A.-H.); Benjamin.tabor@prismahealth.org (B.T.); alexander.milgrom@prismahealth.org (A.M.); joseph.kohn@prismahealth.org (J.K.); 3 Department of Medicine, Division of Infectious Diseases, University of South Carolina School of Medicine, Columbia, SC 29209, USA

**Keywords:** dalbavancin, osteomyelitis, endocarditis, MRSA, bacteremia

## Abstract

The use of long-acting lipoglycopeptides (LaLGPs) in serious, deep-seated infections is of increasing interest. The purpose of this study is to evaluate the economic and clinical utility of LaLGPs in patients requiring protracted antibiotic courses who are not ideal candidates for oral transition or outpatient parenteral antibiotic therapy (OPAT). This is a retrospective, observational, matched cohort study of adult patients who received a LaLGP. Patients were matched 1:1 to those who received standard of care (SOC). Cost effectiveness was evaluated as total healthcare-related costs between groups. Clinical failure was a composite endpoint of mortality, recurrence, or need for extended antibiotics beyond planned course within 90 days of initial infection. There was no difference in clinical failure between the two cohorts (22% vs. 30%; *p* = 0.491). Six patients in the SOC cohort left against medical advice (AMA) prior to completing therapy. Among those who did not leave AMA, receipt of LaLGPs resulted in a decreased hospital length of stay by an average of 13.6 days. The average total healthcare-related cost of care was USD 295,589 in the LaLGP cohort compared to USD 326,089 in the SOC cohort (*p* = 0.282). Receipt of LaLGPs may be a beneficial treatment option for patients with deep-seated infections and socioeconomic factors who are not candidates for oral transition or OPAT.

## 1. Introduction

Dalbavancin and oritavancin are intravenous (IV), long-acting lipoglycopeptides (LaLGPs) with extended half-lives and broad-spectrum of coverage against gram-positive bacteria, including methicillin-resistant staphylococcus aureus (MRSA) [1]. Dalbavancin is approved by the United States Food and Drug Administration (FDA) for acute bacterial skin and skin structure infections (ABSSSIs) as a 1500 mg single dose regimen, or a two-dose regimen of 1000 mg initially followed by 500 mg one week later [2]. Oritavancin is also FDA-approved for ABSSSIs as a single dose of 1200 mg [3].

The LaLGPs were originally marketed to provide a simple, outpatient option to avoid hospitalization in patients presenting to the emergency department and diagnosed with an ABSSSI [4]. The cost effectiveness for this indication is debatable; however, the pharmacokinetic (PK) and dosing advantages offered by LaLGPs have increased interest in use in off-label conditions including serious, deep-seated infections requiring protracted antibiotic courses (e.g., endocarditis, osteomyelitis, bacteremia). Dalbavancin concentrations 2 weeks post a 1000 mg IV dose were sustained well above the minimal inhibitory concentration (MIC)_90_ for *Staphylococcus aureus* in both bone and serum [5]. A PK model of a 2-dose, 1500 mg IV once weekly regimen demonstrated sustained bone and serum concentrations above pharmacodynamic (PD) targets for up to 8 weeks [5]. These findings suggest LaLGPs achieve sufficient concentrations in deep-seated areas and may be an effective treatment option for such infections, rather than solely for use in ABSSIs.

There are limited data examining clinical outcomes and cost savings in patients with osteomyelitis and infective endocarditis who received LaLGPs [5,6,7,8,9]. Although these data are encouraging, the studies are often descriptive in nature, limited to a single hospital, or focused on ABSSSI as an indication. Patients who require prolonged antimicrobial treatment durations and are not candidates for oral therapy or discharge for traditional outpatient parenteral antibiotic therapy (OPAT), such as persons who inject drugs, prisoners, or those experiencing homelessness, may be ideal candidates for LaLGPs. Consideration of these agents may facilitate hospital discharge and be a proactive and targeted antimicrobial stewardship initiative. 

The purpose of this study is to evaluate the clinical and economic utility of LaLGPs in patients requiring protracted antibiotic courses who are not ideal candidates for oral transition or OPAT compared to those receiving standard of care (SOC) in a multi-hospital health system.

## 2. Materials and Methods

This is a retrospective, observational, matched cohort study of adult hospitalized patients who received a LaLGP within the Prisma Health Midlands in Columbia, South Carolina, USA from 1 January 2017 to 1 October 2020. Prisma Health Midlands consists of four hospitals totaling approximately 1500 licensed inpatient beds. The health system Institutional Review Board approved this study prior to initiation. Patients who received a LaLGP were matched 1:1 to those who received SOC therapy by age (±10 years), infection type, microorganism, and presence of at least one socioeconomic factor impacting adherence (e.g., persons who inject drugs, homelessness). The control population was identified via culture information obtained from the microbiology laboratory and electronic health records. One investigator (KA) was responsible for initial screening and case matching, which was confirmed by a second investigator (PBB). SOC therapy was defined as any clinically appropriate agent and duration utilized to treat the designated infection and pathogen (e.g., vancomycin or daptomycin for MRSA).

Patients were included if they were ≥18 years of age, hospitalized in one of the Prisma Health Midlands hospitals, and received dalbavancin or oritavancin (i.e., LaLGP). Enrolled patients were required to: (a) have a clinical diagnosis of infection with gram-positive bacteria and (b) be a poor candidate for oral therapy or unable to be discharged on intravenous antibiotics as determined by the treatment team and case management. Reasons for this may have included but were not limited to lack of financial support, homelessness or inconsistent housing, or documented IV drug use. Patients were excluded if they received a LaLGP for a previous infection or were pregnant.

The primary clinical outcome was clinical failure, defined as a composite endpoint of mortality at 90-days post treatment, recurrence at 90-days post treatment, or need for extended antibiotics beyond the planned course due to lack of clinical resolution. Additionally, each individual component of the composite endpoint was compared between the two groups. Secondarily, the impact on time of hospitalization was also compared, as measured by differences in hospital length of stay and proportion of patients leaving against medical advice (AMA) prior to completing therapy. The primary cost effectiveness outcome was the difference in total health-care related costs between the two cohorts. Line by line patient statements were obtained from the institution billing department for patients in both groups, and raw data were used to calculate the patient’s total healthcare related cost. Drug-specific costs were also calculated and compared. Chi-square or Fisher’s exact tests were used for categorical data and Student’s *t* test, or Mann–Whitney U were used for continuous data. Kaplan–Meier analysis was used to examine clinical failure. Patients lost to follow-up within the 90 days were censored from this analysis. Log-rank *p*-value was used to examine the difference in time to clinical failure between LaLGP and SOC groups. Multivariable analysis was not performed since the two groups were matched based on major demographic, clinical, microbiological, and socioeconomic factors. SAS software (version 9.4) was used for statistical analysis. A two-sided *p*-value of 0.05 was considered statistically significant.

## 3. Results

A total of 46 patients were included in the clinical and economic analysis. Overall, patients had a median age of 45 years, were predominantly Caucasian, *n* = 33 (72%), with even distribution of men and women (23 in each cohort). A history or current use of IV drugs was documented in 56% of patients while inconsistencies in housing, lack of financial support and homelessness were also present. The most frequent indication was endovascular infection, followed by complicated bacteremia and osteomyelitis. The most common organism isolated was methicillin-resistant *Staphylococcus aureus* (MRSA), followed by methicillin-susceptible *S. aureus* (MSSA). No major differences were observed between LaLGP and SOC groups at baseline (Table 1). In the SOC and LaLGP groups, 43.5% and 52.2% of patients, respectively, achieved source control, defined by removal of catheter, drainage of abscess, debridement of infected wounds, valve repair and/or replacement, or any surgical procedure designated to control a focus of infection. 

In the LaLGP group, 22 of the patients received dalbavancin and 1 received oritavancin. The mean number of planned doses of dalbavancin received in the LaLGP group was 1.68 doses, and the majority of patients received two doses (*n* = 15). Of the patients who received multiple doses of dalbavancin, nine received at least one dose in the outpatient infusion center, rather than while hospitalized. The remaining doses were received while patients were hospitalized for their index infection. The most common regimen used was 1500 mg IV × 2 doses administered one week apart. A single missed dalbavancin dose was observed in two patients. For the one patient who received oritavancin, three doses were planned and all three were received in the outpatient setting. 

**Figure 1:**
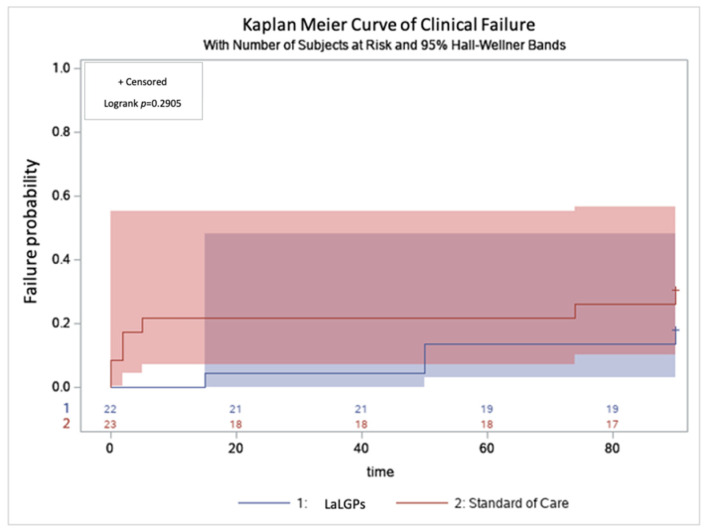
Kaplan–Meier curve of proportion of patients with clinical failure receiving either standard of care or LaLGPs.

The composite clinical endpoint (mortality, recurrence and need for extended therapy within 90 days) was observed in 22% and 30% (*p* = 0.738) of the LaLGP and SOC groups, respectively, and Kaplan–Meier curves for clinical failure were comparable (Figure 1). There were no differences in individual components of the composite clinical endpoint between the groups (Table 2). 

Successful clinical follow up visits were documented in 30% and 13% of patients in the LaLGP and SOC groups, respectively (*p* = 0.284). The mean length of stay was 22.9 days vs. 31.9 days in the LaLGP and SOC groups, respectively (*p* = 0.153). Nearly 26% of patients in the SOC cohort left the hospital AMA prior to completing therapy compared to 0% in the LaLGP cohort (*p* = 0.022). When excluding patients who left AMA, the average LOS was 22.9 and 36.5 days in the LaLGP and SOC groups, respectively. The mean total health-related cost of care was USD 295,589 in the LaLGP group compared to USD 326,089 in the SOC group (*p* = 0.282), resulting in a mean difference of USD 30,500 per patient. A subgroup analysis was conducted after excluding patients who left AMA showing the mean total health-related cost between the two groups was USD 295,589.09 in the LaLGP cohort and USD 351,421.00 in the SOC cohort (*p* = 0.568), resulting in a mean difference of USD 55,831 (Table 3). 

## 4. Discussion

In the present study, LaLGPs showed clinical effectiveness similar to the SOC and clinically significant cost savings among patients with deep-seated infections and barriers to discharge. Receipt of LaLGPs was associated with a mean savings of USD 30,500–55,831 per patient and a cumulative cost savings of USD 701,510 for the entire cohort. Moreover, LaLGPs saved an average of 9 days of hospitalization although not statistically significant likely due to relatively small sample size. Although these results were not statistically significant, this is substantial in terms of institutional cost savings and bed availability for more acute cases, especially during times where institutions are overburdened with an influx of patients as encountered during the current SARS-CoV-2 pandemic. Previous studies showed a similar reduction in length of hospital stay of 9–14 days [8,10]. Secondly, LaLGPs show promise in patients where adherence is questionable, as shown in the patient population studied (e.g., homelessness, persons who inject drugs, patients with financial limitations) [11,12,13]. No patients left AMA in the LaLGP cohort compared to 26% in the SOC cohort, emphasizing the advantages of use of LaLGPs in this patient population and ability for such patients to complete the total intended duration of therapy. Oral regimens for these invasive infections can require multiple-daily dosing and may be associated with increased toxicity in comparison with a LaLGP [14,15]. OPAT has substantial risks as well including peripherally inserted central catheter (PICC) placement and frequent weekly monitoring, which such patient populations may not be able to endure [16]. PICC infectious complications are described in the literature in ~6% to 9% of patients receiving intravenous catheters [14,17]. Other associated risks include occlusions, venous thrombosis, and hematomas [17]. A study analyzing outcomes related to PICC placement in patients with substance use disorders noted drug relapse within 40% of patients labeled “high risk” (injection use within 12 months) [18]. Additionally, the requirements of OPAT (such as consistent housing, water, appropriate refrigeration, power, proper finances) are barriers that many of these patients included in this study may not have access to. Furthermore, it is challenging to account for the cost of readmissions in patients that leave AMA, thus cost savings may even be greater in the LaLGP group than predicted here.

One additional concern in this vulnerable population is the follow-up with infectious diseases specialists post discharge. In our study, the overall follow-up was low, however, it was not negatively impacted by using a LaLGP. It is likely that socioeconomic factors contributed to this much lower clinic follow up rates as compared to the general clinic population. Completing therapy and reducing the proportion of patients who leave AMA may have enhanced the follow-up. Institutions should stress post-discharge follow-up among these patients including contact information and management of additional socio-economic barriers (e.g., transportation, housing). Transitions of care is often a challenge for many institutions, especially among those patients with complex infections. Leveraging the expanding ambulatory stewardship practice may be an opportunity for institutions to help facilitate and enhance the ability to conduct LaLGP and related therapeutic options. 

All but one patient in the present study received dalbavancin as the LaLGP. Our institution transitioned to dalbavancin preferentially due primarily to drug acquisition costs and evolving data for off-label use in deep-seated infections. The average wholesale cost of a 500 mg vial of dalbavancin was around USD 1800 at the time of this writing. The 1500 mg 2-dose regimen frequently used for deep-seated infections requiring protracted courses in the study cohort equates to a cost of nearly USD 10,000 for both doses, which can be a barrier for some institutions and patients. In addition, the majority of patients in this study were uninsured with limited or no consistent source of income. Of note, reimbursement for LaLGPs varies based on site of administration (i.e., inpatient versus outpatient) [9]. A stewardship-led initiative at our institution to assist in transitions of care for patients receiving LaLGPs includes the proactive use of a patient assistance program (PAP) available from the manufacturer (AbbVie Inc.^®^, North Chicago, IL, USA), where medication vials may be sent directly from the company at no cost for qualifying patients [19]. Pizzuti and colleagues demonstrated a cost-benefit using PAPs in patients receiving LaLGP for primarily ABSSSIs [9]. In the present study, only 9 patients received a portion of their LaLGP therapy outpatient. A shift to LaLGP administration to the infusion center upon discharge can facilitate earlier discharge and further offset potential costly medication acquisition price or those associated with longer hospitalizations. However, ensuring that patients will reliably follow-up in the infusion center is important and keeping the patient hospitalized for an additional week may outweigh the benefits of further reduction in hospitalization. Our study did show that despite acquisition costs of dalbavancin, earlier discharge with administration of a LaLGP can still provide overall institutional cost savings for patients with deep-seated infections and increase bed availability. 

A strength of this study was the use of line-by-line bills from the health system billing department, rather than calculating cost estimates. However, these bills take into account what the patient was billed, rather than the actual cost the institution was charged, so costs may be inflated, but to a comparable degree between the two groups. A limitation is low proportion of follow up among patients at the outpatient clinic. All patients followed by the infectious diseases inpatient service had scheduled outpatient follow up visits upon discharge. It is assumed that if a patient experienced clinical failure, they would follow up in clinic addressing their primary concern. However, the lack of follow up makes it challenging to truly determine the clinical failure rate, yet previous studies show similar outcomes data in terms of effectiveness [8]. It is unclear whether clinical failure was due to reinfection or relapse of the current infection, given the high-risk patient population. Complications associated with PICC placement were not assessed in the SOC arm but could offer an additional benefit for patients receiving LaLGPs. Additional limitations include the retrospective nature of the study, as well as the small sample size. Although there was a small sample size, similar, if not more impactful, economic outcomes would be expected with a larger sample size. Additionally, these economic outcomes are relevant in the United States and may not be comparable in other countries. 

## 5. Conclusions

LaLGPs were associated with a numerical, though not statistically significant cost savings, reduced length of stay, and comparable treatment outcomes versus SOC in a cohort of patients with deep-seated, gram-positive bacterial infections. The receipt of LaLGPs may be an effective treatment option used in transitions of care for patients with socioeconomic factors and deep-seated infections who are not candidates for oral transition or OPAT.

## Figures and Tables

**Table 1:** Baseline characteristics of patients with deep-seated gram-positive bacterial infections.

	Standard of Care (*n* = 23)	LaLGP (*n* = 23)	*p*-Value
Age, years (mean ± SD)	47.4 ± 13.8	43.8 ± 13.8	0.374
Sex			0.376
Male	13 (56.5)	10 (43.5)	
Female	10 (43.5)	13 (56.5)	
Race			0.335
Non-Hispanic Caucasian	16 (69.6)	17 (73.9)	
Non-Hispanic African American	4 (17.4)	5 (21.7)	
Hispanic	3 (13.0)	0 (0)	
Other	0 (0)	1 (4.3)	
Socioeconomic factor			
History of injection drug use	13 (56.5)	14 (60.9)	0.765
Homelessness	0 (0)	3 (13)	0.233
Poor candidate for oral therapy or home OPAT	10 (43.5)	7 (30.4)	0.360
Other	1 (4.3)	2 (8.7)	1.000
Positive urine drug screen on admission	8 (34.8)	11 (47.8)	0.512
Charlson Comorbidity Score, median	1 (IQR = 3)	1 (IQR = 3)	0.909
Highest level of care			0.514
Floor	15 (65.2)	17 (73.9)	
Step down unit	0 (0)	1 (4.3)	
ICU	8 (34.8)	5 (21.7)	
qSOFA, median	1.0 (IQR = 1.5)	1.0 (IQR = 2)	0.843
Infection type			1.00
Complicated bacteremia	8 (34.8)	8 (34.8)	
Infective endocarditis	9 (39.1)	9 (39.1)	
Osteomyelitis	5 (21.8) *	5 (21.8) *	
Septic arthritis	1 (4.3)	1 (4.3)	
Microbiology			0.770
MRSA	12 (52.5)	12 (52.5) **	
MSSA	10 (43.5)	10 (43.5)	
CONS	1 (4.0)	1 (4.0)	
Source control achieved	10 (43.5)	12 (52.2)	0.768
Presence of hardware	4 (17.4)	4 (17.4)	1.000

Data are shown as number (percentage) unless otherwise specified. SD: Standard deviation; ICU: intensive care unit; qSOFA: quick sequential organ failure assessment; MRSA: methicillin-resistant *Staphylococcus aureus*; MSSA: methicillin-susceptible *Staphylococcus aureus*; CONS: coagulase-negative staphylococci; AMA: against medical advice; N/A: not applicable; OPAT: outpatient parenteral antibiotic therapy. Chi-square test and Fisher’s exact test (if expected frequency < 5) were used for Categorical data. Student’s *t* test (normally distributed data) and Mann–Whitney U (non-normally distributed data) were used for continuous data. * Vertebral osteomyelitis in *n* = 1 patient for each group ** Based on history and presentation, high clinical suspicion for MRSA in 2 patients.

**Table 2:** Clinical outcomes.

Clinical Outcome	Standard of Care (*n* = 23)	LaLGP (*n* = 23)	*p*-Value
Composite clinical failure	7 (30.4)	5 (21.7)	0.738
90 day mortality	4 (17.4)	1 (4.3)	0.346
90 day recurrence	2 (8.7)	4 (17.4)	0.665
Need for extended antibiotic therapy	3 (13)	4 (17.4)	1.000
Left AMA prior to completing therapy	6 (26.1)	0 (0)	0.022
Length of hospital stay, mean days	32.0 ± 25.0	22.9 ± 19.5	0.153
Excluding those left AMA	36.5 ± 26.0	22.9 ± 19.5	
Clinic appointment follow-up within 90-days of discharge	3 (13.0)	7 (30.4)	0.284

Data are shown as number (percentage) unless otherwise specified. Chi-square test and Fisher’s exact test (if expected frequency < 5) were used for categorical data. Student’s *t* test (normally distributed data) and Mann–Whitney U (non-normally distributed data) were used for continuous data. AMA = against medical advice.

**Table 3:** Economic outcomes.

Economic Outcome	Standard of Care (*n* = 23)	LaLGP (*n* = 23)	Difference, Mean	*p*-Value
Total cost, mean ± SD	USD 326,089.6 ± 227,223.6	USD 295,589.1 ± 350,459.6	USD 30,500.5	0.282
Excluding those left AMA	USD 351,421.0 ± 221,522.6	USD 295,589.1 ± 350,459.6	USD 55,831.9	0.568
Direct cost, mean ± SD	USD 125,292.3 ± 147,397.8	USD 96,471.6 ± 65,674.1	USD 28,820.7	0.475
Total antibiotic cost, mean ± SD	USD 93,893.0 ± 130,755.1	USD 89,131.2 ± 61,682.2	USD 4761.8	0.059

Chi-square test and Fisher’s exact test (if expected frequency < 5) were used for categorical data. Student’s *t* test (normally distributed data) and Mann–Whitney U (non-normally distributed data) were used for continuous data.

## Data Availability

The data presented in this study are available on request from the corresponding author. The data are not publicly available due to institutional policy.

## References

[1] Chen A.Y., Zervos M.J., Vazquez J.A. (2007). Dalbavancin: A novel antimicrobial. Int. J. Clin. Pract..

[2] Dosing. https://www.dalvance.com/about-dalvance/dosing-and-administration.

[3] Dosing and Administration. http://www.orbactiv.com/dosing-and-administration.html.

[4] Bookstaver P.B., Jenkins T.C., Stenehjem E., Doron S., Brown J., Goldwater S.H. (2018). Impact of outpatient vs inpatient ABSSSI treatment on outcomes: A retrospective observational analysis of medical charts across US emergency departments. Open Forum Infect. Dis..

[5] Dunne M.W., Puttagunta S., Sprenger C.R., Rubino C., Van Wart S., Baldassarre J. (2015). Extended-duration dosing and distribution of dalbavancin into bone and articular tissue. Antimicrob. Agents Chemother..

[6] Tobudic S., Forstner C., Burgmann H., Lagler H., Ramharter M., Steininger C. (2018). Dalbavancin as primary and sequential treatment for gram-positive infective endocarditis: 2 year experience at the general hospital of Vienna. Clin. Infect. Dis..

[7] Almangour T.A., Perry G.K., Terriff C.M., Alhifany A.A., Kaye K.S. (2019). Dalbavancin for the management of gram-positive osteomyelitis: Effectiveness and potential utility. Diagn. Microbiol. Infect. Dis..

[8] Morrisette T., Miller M.A., Montague B.T., Barber G.R., McQueen R.B., Krsak M. (2019). Long-acting lipoglycopeptides: “lineless antibiotics” for serious infections in persons who use drugs. Open Forum Infect. Dis..

[9] Pizzuti A.G., Murray E.Y., Wagner J.L., Gaul D.A., Bland C.M., Jones B.M. (2020). Financial analysis of dalbavancin for acute bacterial skin and skin structure infections for self-pay patients. Infect. Dis. Ther..

[10] Arrieta-Loitegui M., Caro-Teller J.M., Ortiz-Pérez S., López-Medrano F., San Juan-Garrido R., Ferrari-Piquero J.M. (2022). Effectiveness, safety and cost analysis of dalbavancin in clinical practice. Eur. J. Hosp. Pharm..

[11] Milgrom A., Bookstaver P.B. (2021). Stewarding the costly antibiotic: Considerations for dalbavancin. Clin. Infect. Dis..

[12] Durante-Mangoni E., Gambardella M., Iula V.D., De Stefano G.F., Corrado M.F., Esposito V. (2020). Current trends in the real-life use of dalbavancin: Report of a study panel. Int. J. Antimicrob. Agents.

[13] Krsak M., Morrisette T., Miller M., Molina K., Huang M., Damioli L. (2020). Advantages of outpatient treatment with long-acting lipoglycopeptides for serious gram-positive infections: A review. Pharmacotherapy.

[14] Li H.K., Rombach I., Zambellas R., Walker A.S., McNally M.A., Atkins B.L., Lipsky B.A., Hughes H.C., Bose D., Kümin M. (2019). OVIVA Trial Collaborators. Oral versus intravenous antibiotics for bone and joint infection. N. Engl. J. Med..

[15] Iversen K., Ihlemann N., Gill S.U., Madsen T., Elming H., Jensen K.T., Bruun N.E., Høfsten D.E., Fursted K., Christensen J.J. (2019). Partial oral versus intravenous antibiotic treatment of endocarditis. N. Engl. J. Med..

[16] Buehrle D.J., Shields R.K., Shah N., Shoff C., Sheridan K. (2016). Risk factors associated with outpatient parenteral antibiotic therapy program failure among intravenous drug users. Open Forum Infect. Dis..

[17] Grau D., Clarivet B., Lotthé A., Bommart S., Parer S. (2017). Complications with peripherally inserted central catheters (PICCs) used in hospitalized patients and outpatients: A prospective cohort study. Antimicrob. Resist. Infect. Control.

[18] Camsari U.M., Libertin C.R. (2017). Small-town America’s despair: Infected substance users needing outpatient parenteral therapy and risk stratification. Cureus.

[19] Patient Assistance, Dalvance. https://www.abbvie.com/patients/patient-assistance/program-qualification/dalvance-program-selection.html#myabbvie.

